# Assessing the roles of population density and predation risk in the evolution of offspring size in populations of a placental fish

**DOI:** 10.1002/ece3.255

**Published:** 2012-07

**Authors:** Matthew Schrader, Joseph Travis

**Affiliations:** 1 Department of Biological Science, Florida State UniversityTallahassee, Florida 32306-1100; 2 Department of Animal Biology, University of IllinoisChampaign, Illinois 61820

**Keywords:** Life history, offspring size, population density

## Abstract

Population density is an ecological variable that is hypothesized to be a major agent of selection on offspring size. In high-density populations, high levels of intraspecific competition are expected to favor the production of larger offspring. In contrast, lower levels of intraspecific competition and selection for large offspring should be weaker and more easily overridden by direct selection for increased fecundity in low-density populations. Some studies have found associations between population density and offspring size consistent with this hypothesis. However, their interpretations are often clouded by a number of issues. Here, we use data from a 10-year study of nine populations of the least killifish, *Heterandria formosa*, to describe the associations of offspring size with habitat type, population density, and predation risk. We found that females from spring populations generally produced larger offspring than females from ponds; however, the magnitude of this difference varied among years. Across all populations, larger offspring were associated with higher densities and lower risks of predation. Interestingly, the associations between the two ecological variables (density and predation risk) and offspring size were largely independent of one another. Our results suggest that previously described genetic differences in offspring size are due to density-dependent natural selection.

## Introduction

Organisms vary tremendously in their investment into individual offspring. Some organisms produce thousands of tiny propagules during a reproductive bout while others produce a few large offspring at one time. This variation has inspired a large theoretical and empirical literature on the evolution of offspring size (reviewed in Roff 1992, 2002; Stearns 1992). In general, the evolution of offspring size will reflect a compromise between the fitness benefits of producing larger offspring and the associated decline in fecundity caused by a trade-off between offspring size and number (Smith and Fretwell 1974; Lloyd 1987). In a constant environment, there will be a single optimal offspring size that balances these costs and benefits. In variable environments, selection might favor producing offspring of varying sizes or the evolution of phenotypic plasticity in offspring size, depending upon the pattern of variation in the selective environment and the existence of reliable cues for the environment a female's offspring will encounter (Kaplan and Cooper 1984; McGinley et al. 1987; Fox et al. 1997; Ghalambor et al. 2007).

The balance between the costs and benefits of producing large offspring will vary with local environmental conditions because the fitness benefits of producing large offspring and the trade-off between offspring size and number may vary with the environment (Parker and Begon 1986; Hutchings 1991; Fox et al. 1997; Fox 2000; Marshall and Keough 2008). The density of conspecifics is an ecological variable that has long been thought to be a major agent of selection on offspring size and indeed this idea is at the heart of an extensive literature in evolutionary ecology (Pianka 1970; Boyce 1984; Reznick et al. 2002; Bassar et al. 2010). The argument is that individuals in high-density populations will experience low resource levels and correspondingly high levels of intraspecific competition, conditions under which larger offspring will be favored (Winn and Miller 1995; Marshall et al. 2006; Bashey 2008). In contrast, low-density populations are likely to be characterized by higher resource levels and selection for large offspring should be weaker and more easily overridden by direct selection for the concomitantly increased fecundity.

Some field studies have found intraspecific associations between offspring size and population density (e.g., Gregersen et al. 2006), suggesting selection in high-density populations has resulted in the evolution of large offspring. However, interpretations of such relationships in natural populations, whether inter- or intraspecific, are clouded by several issues (Mueller 1997). First, few studies of natural populations have measured population densities over a long enough period of time to accurately describe a population as being characteristically high or low density (Mueller 1997). Second, it is often unclear whether variation among natural populations in traits such as offspring size has a basis in genetic variation or whether it reflects phenotypic plasticity (Travis 1994). Third, natural populations differ from one another in many ways and these differences will often be confounded with one another, particularly in surveys of a limited number of populations. Such confounding will complicate the interpretation of an association between density and offspring size or, given the possibility of counter-gradient selection, even the lack of an evident association. As a result, the importance of density-dependent selection as a major force behind the evolution of offspring size in natural populations remains arguable.

Previous work on the relationship between population density and life-history variation in the least killifish, *Heterandria formosa* illustrates the difficulty of settling this issue. In a field study of four *H. formosa* populations (Moore Lake, Trout Pond, Horn Spring, and Wacissa River), Leips and Travis (1999) found that females from the population characterized by the highest density (Wacissa River) produced offspring that were on average 45% larger than offspring from the other three populations. This observation is consistent with selection for large offspring in the high-density population. However, this interpretation is complicated by several issues. First, because population densities were measured in these populations over the course of only two years (approximately six *H. formosa* generations), it was not clear whether the observed density variation was consistent over a long enough time for it to be evolutionarily relevant. Second, it was not clear whether variation among these populations in offspring size had a basis in genetic variation or was due to plasticity in offspring size with population density or some other environmental variable. Third, the differences in population density described by Leips and Travis (1999) were associated with other ecological agents. The two populations with the highest density were cooler, spring-fed rivers with lower predator densities while the two populations with the lowest density were both warmer, lentic ponds with higher predator densities (Leips and Travis 1999). Cooler temperatures and flowing water are associated with larger offspring size in many ectothermic vertebrates (Travis 1994) and predators are well-described selective agents on life-history traits in poeciliid fish (Reznick and Endler 1982; Reznick et al. 1990; Johnson and Belk 2001; reviewed in Johnson and Bagley 2011). The limited scope of Leips and Travis's (1999) initial survey makes the confounding of density with other eco-logical variables particularly problematic.

Subsequent work has resolved some but not all of these complicating issues. For example, there are now two lines of evidence indicating that the density differences observed by Leips and Travis (1999) have persisted over long periods of time. First, studies using microsatellites have demonstrated a positive correlation between average population density estimated during field censuses and average hetero-zygosity among *H. formosa* populations (Soucy and Travis 2003; Schrader et al. 2011). Such a correlation is consistent with long-standing differences in density. Second, Schrader et al. (2011) found consistent and dramatic differences in population density between four *H. formosa* populations censused annually over a 10-year period. This period of time is equivalent to approximately 30 *H. formosa* generations. These results suggest that density-dependent selection is plausible in these populations. Variation among *H. formosa* populations in offspring size has also been shown to reflect genetic variation through both the persistence of population differences in the laboratory (Leips et al. 2000) and the results of direct crosses between populations (Leips et al. 2000; Schrader and Travis 2009).

Despite this progress, the relationship between offspring size and population density has only been assessed in the four populations originally studied by Leips and Travis (1999). A better assessment of the role of population density in driving the evolution of offspring size requires data on densities from a wider range of populations and habitats, along with the concomitant data about other factors that might influence the evolution offspring size. Data on predation risk would be particularly illuminating because predation is a major source of selection on poeciliid life histories (reviewed in Johnson and Bagley 2011). In this study, we use data from a 10-year study of nine *H. formosa* populations to describe the associations of offspring size with habitat type, population density, and predation risk.

## Methods

### Study species

**Figure 1:**
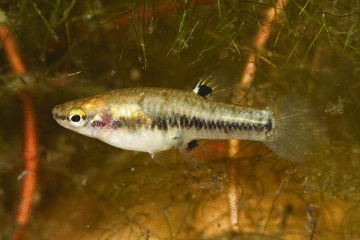
A female *Heterandria formosa*. The female in the photo is approximately 2 cm long. Photo courtesy of Pierson Hill.

The least killifish (Fig. 1), *H. formosa*, is small live-bearing poeciliid fish distributed throughout the coastal plain of the southeastern United States. This species is highly matrotrophic with mothers provisioning embryos between fertilization and birth via a placenta. In addition to displaying a high level of matrotrophy, females provision several broods of embryos simultaneously, a phenomenon known as superfetation. Previous studies of *H. formosa* have described wide variation among a few populations in offspring size at birth (Leips and Travis 1999; Schrader and Travis 2005); differences in offspring size reflect differences in postfertilization maternal investment (Schrader and Travis 2005, 2009). Laboratory studies indicate that differences between some *H. formosa* populations in offspring size have a genetic basis (Leips et al. 2000; Schrader and Travis 2009). However, offspring size is also plastic; females experiencing higher densities and lower per capita resources produce smaller offspring (Leips et al. 2000) but females experiencing higher densities with constant per capita resources produce larger offspring (Leips et al. 2009). The role of food level divorced from density in inducing plasticity is unclear; Travis et al. (1987) found no effect of constant but distinct food levels on offspring size at birth in *H. formosa*, but Reznick et al. (1996) found that *H. formosa* females produced smaller offspring under lower resource levels.

### Field censuses

We estimated population densities and measured female life-history traits in nine North Florida *H. formosa* populations over 10 years. The study populations can be broadly divided into two categories: springs (McBride Slough, Shepherd Spring, Wacissa River, and Wakulla Springs) and ponds (Cessna Pond, Lake Iamonia, Little Lake Jackson, Moore Lake, and Trout Pond). Population densities were estimated twice annually, once in the spring (late April or early May) and again in the late summer (early September). Here, we focus on data from the spring censuses; the September census is after the peak of the breeding season and females collected at this census are often not pregnant (Leips and Travis 1999; J. Travis, unpubl. data).

Population density was estimated using methods described in detail elsewhere (Leips and Travis 1999; Richardson et al. 2006). Briefly, at each census we threw a 0.5 m^2^ throw trap three times in habitat likely to contain *H. formosa* (shallow water with vegetative cover). The contents of each trap throw were removed with 10 sweeps of a dipnet and placed in a water-filled bucket. We then sorted through the contents of each bucket and counted the number of female, male, and juvenile *H. formosa* caught in each trap throw. We also noted the presence of all other vertebrates and invertebrates in each trap throw. All males and juvenile *H. formosa* were returned to the water after they were counted. Whenever possible, we retained 20 females caught during each census for quantification of life-history traits. When we did not capture enough females in the trap throws we attempted to collect additional females using dipnets. During some censuses, we were unable to collect any females in the trap or by dipnetting.

Females collected for the quantification of life-history traits were euthanized with an overdose of MS-222 and preserved in 10% formalin in the field. Females were later measured (standard length in mm) with dial calipers and dissected to determine the number of developing embryos in each developmental stage. Embryos were staged using the classification scheme modified from Reznick (1981). In this classification scheme, stage 2, 3, 4, and 5 embryos correspond to Reznick's (1981) early eyed, mid-eyed, late-eyed, and very late eyed stages (Travis et al. 1987). For each female, we recorded the total number of eyed embryos (fecundity), the number of broods, and the number of embryos in each developmental stage (brood size). We also retained, freeze dried, and weighed broods of stage 4 or 5 embryos. As in previous work (Leips and Travis 1999; Schrader and Travis 2005, 2009), the average dry mass of stage 4 and 5 embryos was used as an estimate of size at birth in each population.

We calculated an index of predation risk for each population in each year by multiplying the incidence of each of five known predator taxa by a measure of each predator's capacity, which is the number of *H. formosa* a single individual predator can capture and consume in 48 h in a standardized predation trial, then summing those products across the taxa detected at that site in that visit (Richardson et al. 2006; Macrae and Travis, in review). The predators were *Lepomis punctatus* (spotted sunfish), *Lepomis gulosus* (warmouth), *Aphredoderous sayanus* (pirate perch), and aeshnid and libellulid dragonfly larvae. Predator incidence was estimated as the number of trap throws at a given census that contained at least one individual predator (this value ranged between 0 and 3). Predatory capacity is derived from data presented in Richardson et al. (2006) for aeshnids (3.62), *A. sayanus* (0.96), *L. gulosus* (10.0), and *L. punctatus* (3.60) (Richardson et al. 2006). We also assigned the values estimated for aeshnids to libellulid dragonflies, which are also capable of capturing and eating *H. formosa.*

## Statistical analyses

### The associations among habitat type and offspring size, fecundity, and predation risk

We first examined whether variation among populations in offspring size and fecundity were associated with broad habitat differences (springs vs. ponds). Offspring size was rarely correlated with maternal size so we chose not to use maternal size as a covariate in the analysis of offspring size. For each census year, we used a nested analysis of variance (ANOVA) to test for differences between habitat types and among populations within habitat type in offspring size. We only included in the analysis collections in which five or more females had stage 4 or 5 embryos. The number of populations included in the analysis varied from year to year due to either the absence of stage 4 or 5 embryos in some collections or failure to collect any pregnant females. The 2008 census coincided with the peak of a long-term drought and we obtained data on offspring size for only one spring and one pond population (Wakulla Springs and Cessna Pond, respectively). For this year, we used a one-way ANOVA to test whether offspring size differed between the two populations.

In superfetating species such as *H. formosa*, a female's fecundity will be determined by the number of broods she is carrying and the number of embryos in each of these broods. For simplicity, we focus here on the total number of eyed embryos carried by a female as a measure of fecundity. This measure of fecundity was positively correlated with the level of superfetation and brood size in all of our populations (*P* < 0.01 in all populations). Preliminary analyses found fecundity to be positively correlated with maternal size in most of our collections so we examined whether fecundity differed between habitats using a two-step procedure similar to that used by Leips and Travis (1999). First, we used an analysis of covariance (ANCOVA) to test whether populations differed in their relationship between maternal size and fecundity. These analyses were performed on each census year separately. In years in which the homogeneity of slopes assumption was not violated (i.e., there was not a significant population by standard length interaction), we tested whether habitat types differed in fecundity using a nested ANOVA with female size as a covariate. The number of populations included in these analyses varied from year to years because we were unable to collect pregnant females from some populations in some years. Fecundity was log-transformed for all of these analyses.

We calculated an average index of predation risk for each population by averaging the predation risk values across years. We then tested whether habitats differed in predation pressure using a *t*-test on the average values for the individual populations found in each habitat.

### The relationships among population density, predation risk, and offspring size and number

Analyses of population density data are presented elsewhere (Macrae and Travis in review). Here, we focus on the relationships between estimates of population density, predation risk, and offspring size. We examined the relationships among population density (log-transformed), predation risk, and each life-history trait independent of broad habitat associations at three levels. First, we tested whether there were correlations between average population density and average offspring size and fecundity within each population across years. Such a correlation would indicate whether these traits vary within a population in response to variation in population density (Leips et al. 2000, 2009). Second, we examined the correlations among population averages for density, predation risk, and each life-history trait, using the averages for all variables in each population taken across all years of the field study. Correlations between average offspring size or fecundity and average population density would be consistent with divergence in female life-history traits in response to density variation. Finally, we employed partial correlations among the population averages to dissect the confounded influences of predation risk and population density on the life-history traits. All correlations are Pearson product-moment calculations.

## Results

### The associations among habitat type and offspring size, fecundity, and predation risk

**Figure 2:**
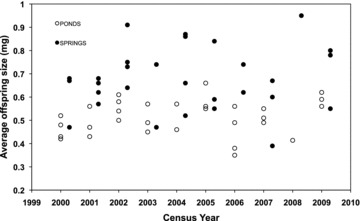
Average offspring size (dry mass of stage 4 and 5 embryos in mg) in spring (closed circles) and pond populations (open circles) censused between 2000 and 2009. Data points are population means.

Offspring size varied tremendously among populations. Average offspring size was largest in the 2008 Wakulla Springs collection (0.95 mg) and smallest in the 2006 Lake Iamonia collection (0.35 mg).There was nearly a 70% difference in offspring size between the population with the highest average offspring size (Wakulla Springs) and the population with the lowest average offspring size (Little Lake Jackson). Although there was considerable variation in average offspring size among populations within both the spring and the pond habitat designations, offspring from spring populations were generally larger than offspring from pond populations. The extent of this difference varied among years (Fig. 2). The largest difference was in 2008 when the average size of offspring from the spring populations was more than double the average size of offspring from the pond populations. The smallest difference occurred in 2009 when the average offspring size from the spring populations was only 14% larger than that from the pond populations. Across all collections, offspring from spring populations were, on average, 37% larger than offspring from pond populations.

Nested ANOVAs on offspring size confirmed these patterns (Table 1). The variation among populations within a habitat type was statistically significant in eight of nine years when a nested ANOVA was possible. Despite such large population variation, there were significant differences between habitat types in three of the eight years (2001, 2002, and 2006). We were unable to examine habitat differences in offspring size in the 2008 collection because we only had measures of offspring size from one spring (Wakulla Springs) and one pond population (Cessna Pond). However, the difference in offspring size between these populations reflects the overall tendency for offspring from springs to be larger than offspring from ponds: Wakulla Springs offspring were significantly larger than Cessna Pond offspring (one way ANOVA, *F*_1, 16_ = 41.66, *P* < 0.0001).

**Table 1:** Results of nested ANOVAs examining the effects of habitat type (springs vs. ponds) and population within habitat type on the dry mass of stage 4 and 5 embryos

Year	Effect	df	*F*	*P*
2000	Pop (Habitat)	5, 108	3.89	0.0028
	Habitat	1, 5	5.03	0.075
2001	Pop (Habitat)	5, 106	2.09	0.072
	Habitat	1, 5	11.00	0.021
2002	Pop (Habitat)	6, 97	2.61	0.022
	Habitat	1, 6	8.90	0.025
2003	Pop (Habitat)	3, 80	10.04	<0.0001
	Habitat	1, 3	0.84	0.43
2004	Pop (Habitat)	5, 86	6.58	<0.0001
	Habitat	1, 5	3.56	0.12
2005	Pop (Habitat)	5, 90	5.01	0.0004
	Habitat	1, 5	0.53	0.50
2006	Pop (Habitat)	4, 71	3.71	0.0085
	Habitat	1, 4	10.42	0.032
2007	Pop (Habitat)	3, 59	10.96	<0.001
	Habitat	1, 3	0.04	0.85
2009	Pop (Habitat)	6, 86	3.66	0.0028
	Habitat	1, 6	1.12	0.33

**Figure 3:**
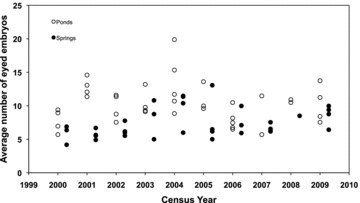
The average fecundity of females in spring (closed circles) and pond populations (open circles) censused between 2000 and 2009. Data points are the least squares mean fecundity for each population after adjusting for female size.

Average fecundity (adjusted for female size) also varied widely among populations (Fig. 3). Fecundity was highest in the 2004 Lake Iamonia collection (19.9 embryos) and lowest in the 2000 Wacissa River collection (4.2 embryos). There was nearly a two-fold difference in average fecundity between the population with the highest average fecundity (Moore Lake, 12.13 embryos) and the population with the lowest average fecundity (Wacissa River, 5.7 embryos). There was considerable variation in average fecundity among populations within both the spring and the pond habitat designations (Fig. 3). Springs always had lower average fecundities than ponds but the large variation in average fecundity among populations within a habitat produced considerable overlap between the values for each habitat in every year but 2001. In 2001, females from ponds had almost three times the average fecundity as females from springs.

Analyses of covariance revealed significant population by female size interactions in four of the 10 years (2001, 2003, 2005, and 2007; Table 2), so we excluded these years when we examined fecundity with nested ANOVAs. We also excluded 2008 from the nested ANOVAs because we only collected females from one spring population in this year. There was a significant effect of habitat type on fecundity in only one of the five years in which a nested ANOVA was possible (2002). In this year, females from pond populations were 54% more fecund, on average, than females from spring populations. There were significant differences in fecundity among populations within habitat type in most years and a significant effect of female size on fecundity in all years (Table 2).

**Table 2:** Results of nested ANOVAs (2000, 2002, 2004, 2006, and 2009) examining the effects of female size (ln female standard length), habitat type (springs vs. ponds), and population within habitat type on female fecundity (ln total number of eyed embryos) or ANCOVAs (2001, 2003, 2005, and 2007) examining the effects of population, female size, and their interaction on female fecundity

Year	Effect	df	*F*	*P*
2000	Pop (Habitat)	5, 132	3.33	0.0073
	Habitat	1, 5	2.48	0.18
	SL	1, 132	26.12	<0.0001
2001	Pop	7, 138	10.65	<0.0001
	SL	1, 138	51.18	<0.0001
	Pop × SL	7, 138	10.44	<0.0001
2002	Pop (Habitat)	6, 151	3.03	0.0079
	Habitat	1, 6	10.53	0.018
	SL	1, 151	35.51	<0.0001
2003	Pop	6, 91	2.76	0.016
	SL	1, 91	33.15	<0.0001
	Pop × SL	1, 6	2.58	0.024
2004	Pop (Habitat)	7, 132	11.91	<0.0001
	Habitat	1, 7	1.31	0.29
	SL	1, 132	13.10	<0.0001
2005	Pop	6, 105	2.34	0.036
	SL	1, 105	26.82	<0.0001
	Pop × SL	1. 6	2.18	0.051
2006	Pop (Habitat)	6, 93	3.85	0.0018
	Habitat	1, 6	0.02	0.89
	SL	1, 93	67.48	<0.0001
2007	Pop	5, 80	2.44	0.042
	SL	1, 80	54.31	<0.0001
	Pop × SL	5, 80	2.40	0.045
2009	Pop (Habitat)	7, 134	4.54	0.0001
	Habitat	1, 7	1.18	0.31
	SL	1, 134	239.55	<0.0001

**Figure 4:**
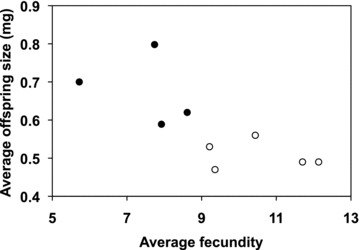
The trade-off between offspring size and number among nine *Heterandria formosa* populations. Solid circles indicate spring populations and open circles indicate pond populations.

There was evidence for a strong trade-off between average offspring size and average fecundity among populations (*r* = –0.73, *P* = 0.026, *N* = 9). Spring and pond populations occurred at different positions along this trade-off: females from spring populations had large offspring and low fecundity relative to pond populations (Fig. 4).

**Figure 5:**
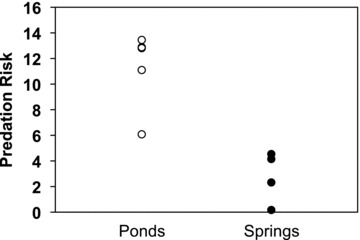
The average predation risk values for pond and spring *Heterandria formosa* populations.

The average predation risk for ponds was nearly five times higher than that of springs (Table 3; Fig. 5). This difference was statistically significant (*t*_7_ = 4.79, *P* = 0.002).

**Table 3:** Average adult density (number of adults per 0.5 m^2^), offspring size (dry mass in mg), and fecundity (number of eyed embryos) in nine *Heterandria formosa* populations censused between 2000 and 2009. For each variable, we report the average value for all years, the range of observed values, and the coefficient of variation. We also report the Spearman rank correlations between each life-history trait (offspring size and fecundity) and population density (log adult density) across years in each population. Significant correlations (*P* < 0.05) are in bold. Sample sizes correspond to the number years included in each correlation. Sample sizes differ among years and traits because of variation in the presence of late-stage embryos and pregnant females

Population	Density	Offspring size (mg)	Fecundity
Cessna Pond	Mean = 4.89	0.53	9.2
	Range = 0–13.7	0.41–0.58	5.7–11.7
	CV = 89.67	11.9	23.70
		*r* = –0.09 (*n* = 6)	*r* = 0.23 (*n* = 8)
Lake Iamonia	2.62	0.49	11.7
	0–11	0.35–0.56	5.7–19.9
	179.3	19.6	49.80
		*r* = 0.54 (*n* = 4)	*r* = –0.089 (*n* = 5)
Little Lake Jackson	9.14	0.47	9.3
	0.3–34.7	0.38–0.62	7.5–11.4
	132.88	19.0	13.92
		*r* = –0.075 (*n* = 5)	*r* = –0.19 (*n* = 7)
McBride Slough	4.77	0.59	7.9
	0–18	0.39–0.80	5.0–11.5
	133.79	21.30	28.31
		*r* = 0.23 (*n* = 9)	*r* = –0.28 (*n* = 9)
Moore Lake	3.48	0.49	12.1
	1.3–6	0.42–0.59	7.0–15.3
	54.17	11.45	20.08
		***r* = 0.69** (*n* = 9)	*r* = 0.27 (*n* = 9)
Shepherd Spring	3.79	0.62	8.6
	0–16	0.52–0.75	6.3–13.1
	151.22	14.26	30.81
		*r* = 0.13 (*n* = 6)	−0.60 (*n* = 7)
Trout Pond	6.81	0.56	9.3
	1–12.3	0.48–0.66	7.5–12.1
	62.96	11.22	17.11
		*r* = 0.19 (*n* = 7)	−0.56 (*n* = 8)
Wacissa River	46.10	0.7	5.7
	7–113.7	0.55–0.87	4.2–6.6
	75.97	14.14	12.34
		*r* = 0.071 (*n* = 9)	*r* = 0.022 (*n* = 9)
Wakulla Springs	32.23	0.80	7.7
	0–56.3	0.55–0.95	4.9–10.8
	65.32	19.13	27.41
		*r =* 0.46 (*n* = 6)	−0.55 (*n* = 9)

### Correlations among life-history traits, population density, and predation risk

Annual variation in average density within a population was substantially larger than annual variation in offspring size (Table 3). Whereas coefficients of variation for average density were at least 54% and ranged to nearly 180%, the coefficients of variation for average offspring size ranged narrowly between 11% and 21%. Not surprisingly in that light, these variables were usually not significantly correlated with one another. The only exception was in Moore Lake, where offspring size significantly increased with population density (Table 4). Given the small number of annual samples, we are reluctant to conclude much about how offspring size and number vary as a function of population density. However, the estimated correlations between offspring size and population density in the populations that exhibited the highest ranges of densities and offspring sizes (McBride Slough, Wacissa River, Wakulla Springs) were all nonsignificant (Table 3). Average fecundity was more variable across years than average offspring size, with coefficients of variation ranging between 12% and 50% (Table 3). Nonetheless, the correlations between fecundity and density across years were not statistically significant in any population.

**Table 4:** Pearson product–moment correlations among population averages, taken across years, for density, predation risk, offspring size, and fecundity. Values above the diagonal are pairwise correlations; values below the diagonal are the partial correlations between each of the two life-history traits and either density or predation risk, each holding the other constant

	Density	Predation risk	Offspring size	Fecundity
Density		−0.28	0.77*	-0.76*
Predation risk			−0.68*	0.51
Offspring size	0.81***	−0.74**		−0.73*
Fecundity	−0.75**	0.47		

* *P* < 0.05,

** *P* < 0.01,

*** *P* < 0.001.

**Figure 6:**
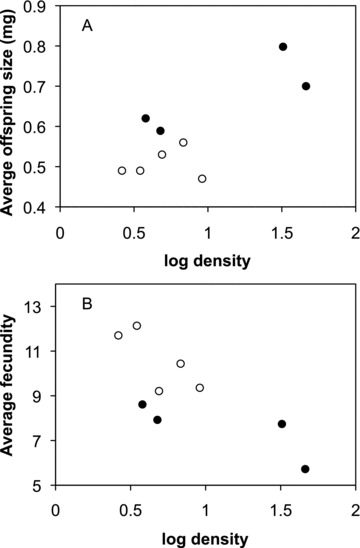
The relationships between (A) average offspring size and log average population density and (B) average fecundity (least squares means) and log average population density in nine *Heterandria formosa* populations. Spring populations are indicated with solid circles and pond populations are indicated by open circles.

**Figure 7:**
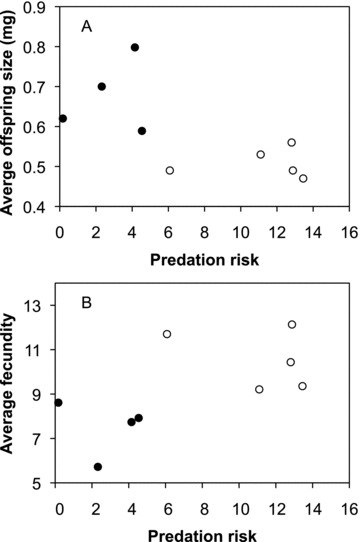
The relationships between (A) average offspring size and predation risk and (B) average fecundity (least squares means) and predation risk in nine *Heterandria formosa* populations. Spring populations are indicated with solid circles and pond populations are indicated by open circles.

Across all populations, larger offspring were associated with higher densities and lower risks of predation. The average population density was positively correlated with average offspring size and negatively correlated with average fecundity (Table 4; Fig. 6). Average predation risk displayed a negative correlation with average offspring size but no significant correlation with average fecundity (Table 4; Fig. 7). The partial correlations between each of the two life-history traits and either density or predation risk, each holding the other constant, are nearly identical to the pairwise correlations (Table 4): density and risk of predation influence offspring size independently of each other.

## Discussion

### Population density and offspring size and number

High-density populations are likely to be characterized by high levels of intraspecific resource competition and this competition ought to select for large offspring. In a previous study of four *H. formosa* populations, Leips and Travis (1999) found that offspring from a single high-density population (Wacissa River) produced offspring that were 45% larger than offspring from three other lower density populations. Based upon this observation, Leips and Travis (1999) suggested that selection in high-density *H. formosa* populations favors the production of large offspring. Our field surveys of nine populations conducted over the course of 10 years provide substantial additional support for this suggestion.

Our field surveys revealed considerable variation among populations in average densities (Table 3; Schrader et al. 2011; Macrae and Travis, in review) and offspring size. The correlation between average offspring size and average population density was positive, consistent with Leips and Travis's (1999) suggestion that selection in high-density populations favors large offspring. This correlation is clearly driven by two populations with very high densities and very large offspring (Wacissa River and Wakulla Springs). The average density of these two populations was more than eight times greater than the average density of the other seven populations and they produced offspring that were 40% larger than the average size offspring from the other seven populations.

While these results are consistent with the pattern predicted if selection in very high-density populations favors the production of large offspring, both of the high-density/large-offspring populations identified in the current study are springs. This raises the possibility that an ecological source of selection other than population density favors large offspring in springs. The association of variation in density with variation in offspring size among the individual spring populations reinforces the interpretation that larger offspring are at least in part an adaptation to consistently high densities. The average density of the high-density springs (Wacissa River and Wakulla Springs) was more than nine times greater than the average density of the low-density springs (McBride Slough and Shepherd Spring) and the average size of offspring from the high-density springs was 24% larger than the average size of offspring in the low-density springs.

We found little evidence that offspring size or fecundity exhibited much plasticity within a population in response to annual variation in population density. Our sample size for each population was between four and nine, so our power to detect correlations in these data is quite limited. However, the apparent absence of plasticity in offspring size in response to population density is at odds with prior experimental results in which higher population densities and lower per capita resource levels were associated with smaller offspring size (Leips et al. 2000) and higher treatment densities with constant per capita food levels produced larger offspring (Leips et al. 2009). There are two possible resolutions to the paradox between our field patterns and the results of previous experiments. First, it is possible that the absence for plasticity in offspring size in the field data reflects a balance between the effects of increased social density (which increases offspring size) and increased resource limitation (which decreases offspring size). This assumes that productivity (grams of carbon available per unit volume of habitat per unit time) is nearly the same in all years such that higher densities always imply lower per capita resource levels. We cannot assess this assumption. Second, it is possible that the plasticity of offspring size with respect to social density is actually a countergradient phenomenon through which offspring size is kept relatively constant in the face of changing resource availability and changing densities. According to this hypothesis, females adjust their investment into individual offspring to the combination of cues reflecting expected crowding and resource levels. While the relatively small annual variation in average offspring size (Table 3) is consistent with this hypothesis, a proper test requires simultaneous manipulation of per capita food levels and social densities.

### Predation risk and offspring size and number

Predation is the perhaps the best-studied source of selection on poeciliid life histories (reviewed in Johnson and Bagley 2011). For example, studies of the Trinidadian guppy, *Poecilia reticulata*, have found that females form high-predation populations mature earlier and have higher reproductive allotments, producing larger broods of smaller offspring, than females from low-predation populations (Reznick and Endler 1982, Reznick et al. 1990). This pattern of life-history divergence has a genetic basis and is consistent with predictions from life-history theory (reviewed in Reznick and Travis 1996). Our field surveys revealed that predation pressure was significantly higher in pond populations of *H. formosa* than in spring populations (see also Macrae and Travis, in review), consistent with previous studies of a smaller number of populations (Leips and Travis 1999; Richardson et al. 2006). If the different predation regimes of ponds and springs are associated with differential selection on *H. formosa* life histories, then we would expect females from pond populations, which have higher average predation pressure, to mature earlier, have higher reproductive effort, and produce large broods of small young compared to females from spring populations.

Our field censuses did not allow us to quantify age (or size) at maturity or reproductive effort. However, our measures of offspring size and fecundity in each population allow us to assess whether variation in these traits with predation pressure is consistent with the pattern observed in other poeciliids. If we simply categorize ponds as high-predation populations and springs as low-predation populations, we find weak support for the hypothesis that offspring size and fecundity have evolved in response to variation in predation regime. When each year was considered separately, there was evidence that females from springs, which experience relatively low-predation pressure, produced significantly larger offspring than females from ponds in three years. Fecundity differed significantly between ponds and springs in only one year, however, the direction of the difference was consistent with the pattern observed in other poeciliids: females from ponds (high-predation populations) had higher average fecundity than females from springs (low-predation populations).

Categorizing ponds as high predation and springs as low predation ignores variation in predation risk within each habitat (see Fig. 5). A more precise vehicle for examining the importance of predation risk within each habitat is to estimate the correlations between average predation risk values and life-history traits. The predation risk values reflect controlled estimates of attack and consumption rates combined with actual estimates of predator density via incidence rates (Richardson et al. 2006), which offer a precision in estimating risk not available in many other studies. We found that average offspring size was significantly negatively correlated with predation risk, while fecundity was positively, although not significantly, correlated predation risk. The direction of these correlations is consistent with the pattern observed in other poeciliids: females from high-predation habitats produce larger broods of smaller young than females form low-predation habitats (reviewed in Johnson and Bagley 2011).

Variation in both predation risk and population density is expected to influence the evolution of life-history traits. In nature, however, these two factors are often tightly linked with one another since high-predation rates act to decrease population density (Reznick et al. 2001; Richardson et al. 2006). In this study, we found a negative although nonsignificant, correlation between population density and predation risk. The absence of a strong negative relationship between these variables is notable for two reasons. First, it indicates that factors other than predation contribute to regulating population density in some populations. Second, it allowed us to use partial correlations to show that each of these environmental variables is correlated with offspring size independently of the other.

### Caveats and conclusions

We have focused on local variation in population density and predation risk as potential sources of selection for offspring of different size in different *H. formosa* populations. Given the correlative nature of our study there are a few important caveats to keep in mind. First, we cannot rule out the existence of other ecological variables that may vary among these populations and that may influence selection on offspring size. For example, some studies have suggested that selection in low-productivity environments may favor the evolution of large offspring (e.g., Johnston and Leggett 2002). This hypothesis, applied to our populations, would predict low productivity in the Wacissa River and Wakulla Springs populations (which have the largest offspring). However, these two populations also display the highest densities of *H. formosa*, an observation that would be difficult to explain if they were also characterized by the lowest productivity. We do not have estimates of primary productivity in our study populations; however, examining the interplay between population density and primary productivity is an exciting avenue for future research. Second, we have argued that variation among *H. formosa* populations in offspring size represents an adaptive response to different regimes of population density and/or predation pressure. We stress, however, that our results are correlative and a more direct test of whether variation among populations in offspring size is adaptive will require either estimates of selection on offspring size in the wild or measures of offspring performance in different environments (e.g., Fox and Mousseau 1996; Fox 2000; Marshall and Keough 2008). Such studies represent a major logistical hurdle in our system. However, it may be possible to estimate selection on offspring size under different density regimes using either mesocosm or competition experiments (e.g., Bashey 2008).

Despite these caveats, our results offer new insight into the ecological forces driving reproductive incompatibilities between *H. formosa* populations. In previous studies involving four *H. formosa* populations (Schrader and Travis 2008, 2009), crosses between a female from a population characterized by small offspring (Moore Lake and Trout Pond) and a male from a population characterized by large offspring (Wacissa River and Wakulla Springs) resulted in a higher rate of aborted embryos than the reciprocal cross or either within population cross. The “large-offspring” populations used in these previous studies are also the populations characterized in the current study by the highest average population densities. Our results suggest that density-dependent selection for large offspring may be driving maternal-fetal coadaptation in *H. formosa* populations and that crosses between populations adapted to different density regimes disrupt this coadaptation.
